# Symbolic scarcity and epistemic waste: a systems theory of academic publishing failure

**DOI:** 10.1186/s41073-026-00193-3

**Published:** 2026-03-12

**Authors:** Trym Hansen

**Affiliations:** University of Southeastern Norway, Kongsberg, Norway

**Keywords:** Academic publishing, Peer review, Epistemic waste, Novelty bias, Replication crisis, Generative AI in science

## Abstract

**Background:**

Contemporary academic publishing increasingly operates under conditions of symbolic scarcity, shaped by rising submission volumes, constrained reviewer capacity, and evaluative incentives that privilege novelty, visibility, and perceived impact. While these dynamics vary across disciplines, their cumulative effect is a publication system that systematically favors novel and attention-generating contributions over replication, confirmation, and cumulative refinement. As a result, methodologically sound research that tests, stabilizes, or contextualizes existing findings is more likely to be delayed, displaced, or rendered invisible, contributing to persistent non-replication and long-term fragility of the scientific record.

**Methods:**

This paper develops a systems-theoretic analysis of academic publishing, examining how prestige filtration, redundancy in peer review, and novelty-oriented selection interact with institutional incentive structures. Rather than attributing dysfunction to individual actors or emerging technologies, the analysis focuses on how current publication architectures shape evaluative behavior, reviewer labor allocation, and the composition of the visible scientific record.

**Results:**

The analysis identifies three interlocking failure modes: (1) a structural decoupling between epistemic contribution and publication outcomes driven by novelty-oriented symbolic selection; (2) systematic waste of reviewer labor due to non-transferable and repetitive evaluation processes; and (3) declining scalability of existing review infrastructures as generative AI lowers the cost of manuscript production while increasing evaluative load. Together, these dynamics suppress replication and cumulative verification, distort the visible scientific record, and misdirect expert attention away from epistemically stabilizing review.

**Conclusion:**

To address these failures, the paper proposes a two-tiered publishing architecture that separates epistemic inclusion from symbolic curation, alongside a complementary tiered review model that aligns review intensity with epistemic risk. An optional framework for reviewer recognition is also outlined to support sustained evaluative engagement without undermining anonymity. These proposals are offered as conceptual system designs rather than prescriptive reforms, intended to clarify how current publishing architectures generate epistemic waste and to suggest structurally feasible pathways toward a more coherent, inclusive, and resilient scholarly communication system.

## Introduction

The academic publishing system, originally designed to disseminate and verify scientific knowledge, now operates within an environment shaped by symbolic prestige, institutional path dependence, and increasing evaluative pressure. What was once a mechanism for integrating reliable findings into the scholarly record has gradually become a competitive filtration process, with highly selective journals often prioritizing reputational coherence and visibility. Although such dynamics vary across disciplines, the prominence of impact metrics and brand signaling has influenced publication trajectories in ways that are only partially aligned with epistemic contribution [2, 9].

These developments create challenges that are not only distributive but epistemological. As researchers and institutions rely heavily on symbolic indicators of quality, scientific work may be assessed as much for its perceived potential to attract attention as for its substantive contribution to cumulative understanding [6, 11]. Reviewer labor, typically voluntary, frequently redundant, and largely anonymous, supports this system but lacks mechanisms for recognition or reuse [1]. The emergence of generative AI further amplifies these pressures by lowering barriers to manuscript production and increasing submission volume beyond the capacity of existing review workflows [4]. The result is an evaluative infrastructure that is increasingly strained, and whose symbolic incentives do not always align with epistemic needs.

This paper offers a structural analysis of these challenges and proposes a systems-theoretic framework for redesigning publication and review processes. We argue that current incentives create three persistent forms of misalignment: (1) the decoupling of contribution from publication, where novelty and selectivity may overshadow methodological soundness; (2) the inefficient use of reviewer effort due to limited transferability, opacity, and the absence of consistent recognition; and (3) the lack of scalability under conditions of AI-accelerated content production. To address these issues, we outline two integrated architectures: a tiered publishing model and a tiered review system that aim to broaden epistemic inclusion, reduce redundancy, and support more efficient allocation of expert attention.

By examining prestige-driven filtering and modeling alternatives that separate validation from visibility, we aim to clarify how publication systems might adapt to emerging pressures while preserving the reliability of the scientific record. Scientific progress depends on the stability and inclusiveness of this record, and systems that restrict entry based on criteria only loosely connected to epistemic merit risk distorting the knowledge base. The reforms proposed here are therefore not presented as prescriptive solutions, but as conceptual models intended to support more sustainable and coherent forms of scholarly communication across diverse research contexts.

## Structural critique of the prestige-rejection paradigm

The contemporary academic publishing system often departs from the ideals of epistemic openness and knowledge efficiency, particularly in high-impact outlets. Structural incentives related to symbolic scarcity and prestige preservation increasingly influence editorial decision-making. This section examines how some journals have taken on status-maintaining functions, where selection criteria may reflect institutional legitimacy, citation expectations, and brand positioning alongside epistemic contribution and methodological soundness. We analyze the role of rejection as a symbolic filter, the impact factor as a reputational signal, and the emerging divergence between contribution and publication across different segments of the publishing landscape.

### Rejection as symbolic scarcity

Rejection remains a central mechanism through which many selective journals maintain their prestige. While peer review is formally framed as a process for ensuring methodological rigor, in highly competitive outlets it can also function as a symbolic filter that signals exclusivity. Acceptance rates at many high-impact journals remain under ten percent, not because the majority of submissions lack scientific validity, but because low acceptance rates serve as a recognized marker of selectivity. This exclusivity helps sustain a journal’s position in academic rankings, attracts established authors, and supports subscription models or article processing charges [2].

This form of symbolic scarcity is not epistemically necessary. In a digital publishing environment where distribution costs are minimal and archival space is effectively unconstrained, there is no technical requirement for journals to reject large volumes of sound work. The primary constraint is reputational: selective journals derive part of their status from limiting access, which means that rejection can operate as a performance of value rather than a direct assessment of scientific merit.

Over time, this mechanism has become partially decoupled from its original epistemic function. Reviewers are frequently asked to evaluate not only methodological soundness but also novelty, anticipated impact, and alignment with a journal’s thematic priorities. These additional criteria introduce subjective elements into the evaluation process and may reflect prevailing disciplinary norms or editorial interpretations of scope. As a result, some methodologically sound studies may be rejected not for failing scientific standards, but because they are not judged to enhance the symbolic profile of the journal [9].

These dynamics contribute to an epistemic filtering process that tends to favor research perceived as innovative or disruptive. Foundational, confirmatory, or non-novel work may receive less attention in highly selective venues, even when such work provides valuable contributions to cumulative understanding. In this sense, publication outcomes can sometimes resemble a prestige-allocation process, where visibility depends partly on alignment with symbolic priorities rather than solely on epistemic contribution.

### Impact factor and prestige signaling

The impact factor was originally developed as a bibliometric tool to assist librarians in identifying frequently cited journals. Over time, however, it has become a widely used symbolic currency within academic publishing. Many journals now orient themselves strategically toward increasing their impact factor, and by extension, their perceived prestige. This shift has influenced incentive structures in ways that prioritize articles expected to attract rapid citations over those offering long-term or foundational contributions [6].

These incentives have also shaped editorial judgment. Editors may feel pressure to select papers that are likely to generate citation activity, favoring high-profile topics, controversial findings, or methodologically novel studies. As a result, editorial decisions can be informed not only by scientific merit but also by expectations of citation performance. This shift introduces selection pressures into the academic record by underrepresenting research that does not promise immediate visibility [2].

The symbolic function of the impact factor also influences researcher behavior. Authors increasingly target high-impact outlets not primarily because the venue best fits their intellectual goals, but because publication in such outlets is an institutional requirement for hiring, promotion, or funding. This contributes to a recursive loop: authors tailor their work to satisfy the expectations of prestige journals; journals calibrate their selectivity to maintain their metrics; and institutions reinforce the dominance of journals whose prestige is defined by those same metrics. Within this system, the function of publishing as a mechanism for knowledge dissemination and refinement can become secondary to symbolic optimization.

These incentive structures shape an environment in which strategic considerations may at times supersede epistemic ones. While many journals support systematic reviews and meta-analyses due to their citation frequency, other forms of cumulative or confirmatory work may receive less attention, particularly when they fall outside citation-dense research clusters. The result is a publication ecosystem that can become self-reinforcing and status-preserving, in ways that do not always align with exploratory or truth-maximizing scientific goals.

### Contribution without publication

A notable paradox of the current publishing landscape is that contribution and publication can become structurally decoupled. It is increasingly possible for researchers to produce work that is methodologically sound, theoretically coherent, and epistemically valuable, yet struggle to publish it. This pattern is often not due to lack of scientific merit, but to the narrow and competitive selection filters of prestige-oriented journals.

Because these journals are constrained more by symbolic scarcity than by technical limitations such as bandwidth or storage, they are structurally incentivized to reject more papers than they accept. This dynamic can lead editors to prioritize submissions that reinforce the journal’s identity or are perceived as highly novel, even when other submissions are scientifically robust [11].

This contributes to a growing archive of unpublished but methodologically sound research; an epistemic blind spot that shapes what becomes visible as “the scientific record.” Such work may circulate informally, reappear in revised or diluted forms, or move through multiple rounds of submission to lower-ranked outlets. The cost of this pattern is not only the waste of reviewer and author effort, but also the delayed integration of useful findings, replications, and methodological refinements that become available only after prolonged symbolic filtering.

At scale, this dynamic skews the visible body of scientific knowledge. Because publication remains the threshold for recognition and citation, only the subset of research that passes through selective filters becomes part of the formal record. Contribution without publication therefore becomes an increasingly recognizable structural feature of contemporary science, reflecting a system organized around reputation and symbolic positioning as much as epistemic representation.

## Reviewer labor and the paradox of post-review rejection

Peer review is widely regarded as a cornerstone of academic integrity, yet its current implementation reveals several structural inefficiencies. One example is the rejection of manuscripts *after* full peer review, a practice that, while not universal, represents a non-trivial source of wasted labor within segments of the publishing ecosystem. This section examines the dynamics that lead to post-review rejection and how these dynamics consume expert time, create redundancy across journals, and complicate the epistemic function of peer review.

### The uncompensated cost of review labor

Peer review constitutes a form of highly skilled intellectual labor that is almost universally uncompensated. Academics undertake this work as part of their professional expectations, contributing to the communal infrastructure of science. However, the volume and complexity of manuscripts have increased substantially, while recognition, institutional support, and professional incentives for review labor have not kept pace [1].

When a manuscript is rejected after undergoing full review, some portion of the reviewers’ labor does not translate into a citable or enduring scholarly product. Although authors often benefit from the feedback and many manuscripts improve across resubmissions, the reviews themselves are seldom archived, indexed, or made available to the broader community. When the same manuscript is later submitted elsewhere, editors must initiate a new round of review, frequently involving similarly overextended experts and repeating a substantial portion of the evaluative process. While prior feedback can improve manuscript quality, the structural duplication of effort represents a meaningful opportunity cost in terms of time, attention, and analytical capacity across the research system.

In principle, peer review is intended to ensure that only methodologically robust and conceptually sound contributions enter the published record. In practice, reviewers in selective outlets may also be asked to evaluate novelty, fit, and potential impact. These criteria are subjective and vary widely across journals. This additional evaluative layer can shift the function of peer review from strictly epistemic validation toward a broader symbolic or strategic assessment.

### Review redundancy and the failure of knowledge recycling

The current architecture of academic publishing often results in manuscripts undergoing multiple independent rounds of peer review at different journals. In many cases, reviewers re-evaluate core issues such as methodological rigor, conceptual clarity, and evidentiary adequacy without any formal mechanism for sharing prior assessments. This creates a fragmented evaluative environment in which previous reviews are not systematically preserved or transferred.

This redundancy imposes costs on reviewers, editors, and authors alike. Reviewers may be asked to reassess work that has already been deemed methodologically sound but was rejected due to journal-specific priorities or strategic considerations. Editors must initiate new review processes even when earlier assessments might have provided useful context. Authors often revise or reframe manuscripts not to improve scientific content but to align with the stylistic or thematic expectations of a new venue. While iterative resubmission can enhance a manuscript, the absence of shared evaluative memory means that improvements occur alongside substantial duplication of labor.

Some initiatives such as transferable peer review or shared review pools, attempt to address these inefficiencies, but their adoption remains limited. Without broader implementation, much reviewer labor continues to be ephemeral despite its value to the scientific process.

This variability in review outcomes across journals does not necessarily indicate scientific disagreement but highlights the degree to which editorial decisions are shaped by venue-specific priorities. A manuscript rejected by one journal and accepted by another with minimal changes illustrates that review outcomes can be influenced by institutional context as much as by epistemic criteria.

### The evolving purpose of peer review

In its ideal form, peer review safeguards the quality of the scholarly record by identifying methodological weaknesses, clarifying ambiguous claims, and ensuring alignment with disciplinary standards. Over time, however, the expectations placed on reviewers have broadened. Reviewers at selective journals are sometimes asked to comment on a manuscript’s anticipated “impact,” “novelty,” or “significance.” These terms are often undefined and applied inconsistently, and while they serve legitimate editorial considerations, they can introduce subjectivity into the evaluation process. In most journals, such discussions occur primarily at the editorial level rather than through explicit reviewer instructions, but perceived expectations can nevertheless shape reviewer recommendations.

This expanding evaluative scope can create tension in how reviewers conceptualize their role. Rather than operating solely as evaluators of scientific validity, reviewers may feel compelled to assess a manuscript’s alignment with journal priorities or its potential contribution to the journal’s reputation. In such cases, scientifically sound manuscripts may receive divergent recommendations depending on how reviewers interpret these broader criteria.

At the same time, traditional forms of methodological and theoretical conservatism serve important epistemic functions that can be overshadowed when reviewers are implicitly steered toward evaluating novelty or anticipated impact. Conservative review practices foster reflection on methodological lineage, help preserve awareness of historical contingency, and ensure continuity between emerging findings and established frameworks. When these reflective functions are displaced by symbolic criteria, the system risks losing the slow, integrative work that stabilizes theories over time. Recognizing this dual role is essential: the problem is not conservatism itself, but rather the way prestige-linked expectations can distort or suppress the reflective, historically grounded aspects of reviewer judgment.

These dynamics contribute to the phenomenon of post-review rejection in certain segments of the publishing ecosystem. This pattern is concentrated primarily in highly selective journals, whereas most field-specific or mid-tier venues rarely reject manuscripts after substantive revision unless major methodological issues remain. Manuscripts may be declined not because they fail to meet scientific standards, but because they do not align with a journal’s strategic or symbolic goals. As long as peer review remains entangled with prestige-linked considerations, some reviewer labor will be allocated toward maintaining journal identity rather than solely supporting epistemic evaluation.

## Novelty bias and the replication gap

The modern scientific publishing system frequently prioritizes novelty over replication, confirmation, or cumulative refinement, particularly within high-impact journals. While the emphasis on innovation reflects legitimate values such as intellectual progress, originality, and the advancement of knowledge, it can also create structural imbalances when it becomes the dominant evaluative criterion. In many selective venues, papers that introduce new concepts, models, or empirical patterns are favored over those that test, refine, or replicate existing findings [7]. This dynamic does not characterize all areas of publishing, but it plays a substantial role in shaping the visible scientific record in a number of disciplines.

Novelty bias is rarely codified explicitly in editorial policies, but it can arise indirectly from a combination of reviewer expectations, editorial priorities, and journal branding strategies. In selective outlets, reviewers may interpret requests for “contribution to the field” or “significance” as an emphasis on innovation, even when such criteria are not strictly defined. At journals that seek high citation visibility, editors may prioritize manuscripts with perceived media or citation potential. By contrast, many discipline-specific or methodological journals explicitly encourage replication studies, transparency, and the publication of null findings. These diverse practices highlight that novelty incentives vary widely across the publishing ecosystem.

The consequences of novelty-oriented selection are multifaceted. In some fields, the pursuit of originality can incentivize riskier research practices or the selective framing of data to enhance the appearance of contribution. Methodological conservatism and careful confirmatory work which is essential for ensuring the stability of scientific claims, may receive less attention in journals that prioritize rapid conceptual advancement. This dynamic contributes to an imbalance in the published record, where high-variance, low-replication findings are more visible than cumulative or negative-result studies [3]. At the same time, it is important to note that many journals and subfields are actively working to counteract these tendencies through dedicated replication tracks, registered reports, and open-science reforms.

The replication crises in psychology, medicine, and parts of economics cannot be attributed solely to novelty bias; they also reflect publication biases against negative results, underpowered studies, and methodological heterogeneity. Nonetheless, novelty incentives within high-impact venues play a contributing role. When the perceived value of a study is tied to the appearance of forward motion, cumulative verification work may be deprioritized, affecting the long-term stability of empirical literatures.

A structural consequence of novelty-driven publication incentives is that entire populations can remain epistemically invisible. This is especially evident in medicine, where diagnostic algorithms, risk scores, and prediction models are often trained on data drawn predominantly from white populations. Replication across diverse demographic groups which is methodologically essential for generalizability, is frequently undervalued because it lacks novelty. This has measurable clinical consequences: well-known health-management algorithms underestimate risk in Black patients due to biased proxy measures and insufficient cross-population evaluation [8]. Similar issues arise in clinical tools that rely on evidence bases built from demographically narrow samples [5]. Recent reviews of AI-driven diagnostic systems show that underrepresentation in training datasets leads to reduced predictive accuracy for minority groups, reinforcing health disparities that could be mitigated through systematic replication and validation [10]. These cases demonstrate that undervaluing cumulative, population-specific research is not merely an epistemic inefficiency, it has direct implications for scientific validity and public health.

## Proposal: tiered publishing architecture

The structural critiques outlined in previous sections suggest that many challenges in academic publishing arise not from technological limitations but from symbolic and institutional design choices. Issues such as artificial scarcity, the inefficiencies of reviewer labor, and the misalignment of epistemic priorities are shaped by incentive structures rather than by constraints on dissemination. In this section, we outline a systems-theoretic alternative: a tiered publishing architecture designed to realign dissemination, recognition, and epistemic integrity. The core aim is to separate publication from prestige; ensuring that all methodologically sound research enters the scholarly record while preserving space for curated selectivity where it serves professional and disciplinary functions.

The tiered publishing architecture is grounded in the epistemic commitment that access to the scholarly record should track methodological validity rather than symbolic status. Its normative foundation emphasizes transparency, inclusion, and cumulative coherence: all sound work should enter the archive so that the discipline retains a complete and inspectable record of inquiry. This approach reflects a systems-level view of science as an evolving knowledge commons, where resilience depends on reducing information bottlenecks and ensuring that valid contributions, whether incremental or innovative, remain available for scrutiny, replication, and synthesis. By explicitly separating dissemination from symbolic distinction, the model aligns publication practices with the core values of empirical inquiry rather than with competitive signaling dynamics.

Separating dissemination from prestige also carries sociopolitical implications. By ensuring that all sound work enters the archive, the model reduces the structural disadvantages faced by early-career researchers, scholars outside elite institutions, and researchers in low-resource environments. Broader visibility of valid contributions may shift institutional patterns of recognition, enabling a more diverse and globally distributed set of researchers to participate in cumulative scientific development.

### Epistemic inclusion through integrity-filtered publication

The foundation of the proposed model is the principle that all scientifically valid work should be publishable. Rather than relying on pre-publication rejection to maintain brand identity, journals would adopt a two-layer structure. In the first layer, submissions that meet basic methodological, ethical, and theoretical criteria verified through editorial screening or light integrity review would be published in an open-access venue. This reframes the primary function of journals from symbolic gatekeeping to epistemic vetting.

Prototype models already exist along parts of this spectrum. Platforms such as arXiv, OSF, and F1000Research allow broad dissemination of sound work, sometimes through community-based review or post-publication feedback. However, these platforms remain only partially integrated into the prestige economy of academia. The innovation in the proposed architecture is therefore symbolic rather than technical: normalizing an open publication layer as the standard pathway into the scientific record.

A first-layer publication model would help reduce unnecessary rejection and preserve reviewer labor by ensuring that methodologically sound work becomes part of the citable scholarly archive. It would not eliminate variability in visibility or influence, but it would make the initial inclusion of sound research less contingent on venue-specific priorities.

Importantly, this framework also acknowledges that some studies remain unpublished for reasons beyond elite selectivity. Early-phase drug development provides a clear example: safety studies, dose-finding trials, and discontinued compound investigations are often withheld for commercial reasons despite being methodologically sound. These cases illustrate that non-publication can arise from structural incentives unrelated to journal prestige, just as publication bias against null findings suppresses valuable confirmatory work. The proposed model is therefore meant to complement, rather than replace, broader reforms that address these additional sources of epistemic loss.

### Prestige curation without scarcity

While an open-access publication layer addresses epistemic inclusion, it does not negate the social and professional functions that prestige currently serves. Academic careers, institutional rankings, and funding systems rely on differentiation and selective signaling. The proposed architecture preserves these functions by relocating prestige from the act of exclusion to the act of curation.

In the second layer of the model, journals would continue to select, highlight, and curate subsets of already published research based on criteria such as significance, innovation, methodological rigor, or disciplinary relevance. These curated selections could take the form of editorial highlights, featured collections, or periodic print editions as forms of recognition that maintain symbolic value without preventing other valid work from entering the record.

This shift parallels developments in other content ecosystems. In digital media, broad publication platforms coexist with curated playlists, editorial features, and recommendation systems that confer distinction without imposing scarcity. The distinction remains meaningful, but it is derived from post-publication recognition rather than pre-publication filtration.

To reinforce the separation between epistemic inclusion and symbolic distinction, citation metrics and impact indicators could be calculated based on curated second-layer selections rather than the entire publication pool. This approach reduces incentives to restrict publication volume and encourages journals to curate judiciously from a broad base of validated work.

Under this model, authors gain immediate inclusion of sound work in the scholarly archive, while still retaining the possibility of earning symbolic recognition through the curation layer. Journals retain their role as disciplinary tastemakers, but without serving as bottlenecks in the dissemination of methodological soundness. Prestige becomes a transparent, dialogical process rather than an opaque barrier to entry.

By moving prestige to the level of curation rather than exclusion, journals retain their role as interpreters of disciplinary significance without turning publication itself into a scarce commodity. This approach does not frame prestige as a market-like hierarchy, but as a symbolic layer that operates parallel to the inclusive archive. The purpose of curation is epistemic and interpretive rather than economic: to highlight contributions that shape disciplinary trajectories while ensuring that all methodologically sound work is available for scrutiny, replication, and cumulative refinement. In this model, differentiation does not determine access to the record; it determines the visibility of ideas within it.

## Proposal: tiered review architecture

The integrity of scientific publishing depends not only on how knowledge is disseminated but on how it is evaluated prior to dissemination. Existing review practices already vary widely across fields, including double-blind and single-blind review, open peer commentary, editorial-only screening, registered reports, and community-based models.

Despite this diversity, many journals still treat all submissions as if they require the same level of scrutiny regardless of claim strength, novelty, or epistemic risk. This undifferentiated approach leads to reviewer fatigue, delays in publication, and inefficient use of cognitive labor.

The tiered review architecture proposed here builds on existing practices by aligning the depth of review with the epistemic function of the submission. Rather than treating all papers as potential paradigm shifts requiring expert judgment, this model introduces a stratified approach: some papers are reviewed for soundness and coherence, others for originality and impact.

The tiered review architecture is anchored in the principle that evaluative scrutiny should scale with epistemic risk. Rather than assuming that all manuscripts demand identical forms of judgment, this model recognizes that different types of claims require different kinds of expertise, depth, and methodological caution. Its normative foundation lies in proportionality, clarity, and responsible allocation of cognitive labor: review efforts should be concentrated where the potential for conceptual distortion or empirical error is highest. This approach treats review not as a gatekeeping ritual but as a structured epistemic function aimed at preserving the reliability, interpretability, and long-term stability of the published record.

A tiered review system also has implications for the social organization of scholarly labor. By aligning reviewer effort with epistemic risk, the model reduces the hidden inequities of current practice, in which early-career researchers and scholars outside core networks often face disproportionate rejection rates while senior experts carry unsustainable evaluative burdens. More predictable and proportionate review pathways may foster a fairer distribution of cognitive labor and strengthen participation across the research community.

A tiered review structure necessarily involves different levels of evaluative responsibility, but these differences are epistemic rather than social. Reviewers at higher tiers are not elevated by status or professional rank; they are simply those whose domain expertise is required for assessing claims that carry greater theoretical scope, methodological complexity, or epistemic risk. In this model, hierarchy is functional, not symbolic: it exists to ensure that manuscripts receive the level of scrutiny appropriate to their claims, not to create classes of researchers or reviewers. Because such functional hierarchy is an unavoidable part of rigorous scientific evaluation, the purpose of the system is not to eliminate hierarchy but to prevent it from drifting into prestige-based stratification.

The result is a system that is faster, fairer, and more scalable, particularly in the face of accelerating submission volumes.

### Claim-based review thresholds

In the current system, all submissions are routed through the same review protocol, whether they present high-stakes theoretical interventions or routine replications. This uniform approach creates a bottleneck in labor distribution and rests on the assumption that all contributions pose comparable epistemic risk. A tiered model addresses this limitation by matching review intensity to the type of claim being made.

Papers that make incremental, confirmatory, or applied contributions, and that do not attempt to overturn prior knowledge or establish new theoretical frameworks, would undergo soundness review. This involves assessing whether the methodology is appropriate, the analysis internally coherent, and the conclusions proportionate to the presented evidence. These assessments can be carried out by trained graduate students, early-career researchers, staff reviewers, or others with verified domain competence.

By contrast, submissions that introduce new theoretical constructs, challenge disciplinary consensus, or offer radical reinterpretations require full expert review. These papers demand deeper scrutiny of assumptions, implications, and cross-disciplinary connections, and therefore call for the judgment of experienced specialists whose expertise is most valuable in high-risk, high-impact evaluations.

Although these tiers differ in function, they do not create fixed reviewer categories. The distinction is based on claim-dependent epistemic risk rather than academic rank or status. Reviewers may work across both tiers depending on their expertise, methodological familiarity, and the nature of the submission. In this structure, soundness review and expert review operate as complementary components of a unified evaluative system, each preserving a different dimension of epistemic integrity without establishing permanent reviewer classes.

A tiered structure also clarifies the allocation of scarce expertise. Senior domain experts possess specialized knowledge that is most effective when directed toward submissions that introduce conceptual innovation or carry significant interpretive stakes. Placing routine soundness checks in the hands of qualified early-career or mid-career reviewers does not diminish their importance; it ensures that the system reserves high-level disciplinary judgment for the manuscripts that genuinely require it. This is essential for maintaining rigorous evaluation of the most complex and consequential work, including papers considered for curated or featured publication within a journal.

This model therefore introduces a structured form of cognitive triage. It recognizes that submissions differ in epistemic weight and that aligning review depth with claim type strengthens the overall evaluative process. By allocating review labor with greater precision, the system reduces unnecessary delays while preserving the quality of expert adjudication where it matters most.

### Compensating review labor where it matters

The academic review system depends on a vast and mostly invisible layer of cognitive labor. While senior reviewers contribute for reasons of legacy, identity, and intellectual stewardship, the majority of review volume occurs at the lower levels of epistemic risk.

These reviews are critical, but they do not require the judgment of disciplinary architects. They require attention, training, and time, all of which are increasingly scarce among established scholars, yet abundant in untapped academic labor pools.

A tiered review architecture makes it possible to disentangle epistemic evaluation from symbolic obligation, and in doing so opens the door to scalable compensation. Reviewers performing first-layer evaluation, focused on internal consistency, adherence to ethical and methodological norms, and alignment with journal scope, could be drawn from a broader pool of qualified contributors.

For many early-career researchers or independent scholars, reviewing represents not only service but professional opportunity. Compensation in this context is not a commodification of science. It is a mechanism for capacity expansion and institutional coherence.

Micro-payments, fellowships, or review-based stipends can turn review into a legitimate micro-employment stream. These reviewers also gain exposure to evaluative criteria, methodological diversity, and the dialogic nature of peer review, experiences that accelerate their development as researchers.

This model does not eliminate symbolic review. It clarifies where symbolic labor is sustainable and where it must be supplemented by economic realism. At the same time, it must avoid inadvertently creating a new prestige hierarchy between compensated and uncompensated reviewers. Participation at different tiers must reflect epistemic roles rather than status.

### Reviewer recognition as epistemic capital

The academic publishing system relies on a relatively small and consistently engaged group of scholars to carry out its most demanding evaluative work. These individuals, often senior academics or experienced disciplinary contributors, undertake a substantial share of peer review not because they receive compensation, but because they view the activity as an integral part of scholarly practice. Reviewing has long been associated with maintaining standards within a discipline and contributing to the quality and continuity of the scientific record.

However, the conditions under which this work occurs have shifted. Submission volumes continue to rise, interdisciplinary research has grown more complex, and the time available to established scholars has become increasingly constrained. Many of those who provide high-quality reviews also balance extensive teaching, administration, supervision, funding responsibilities, and their own research agendas. Within these competing demands, review work is often fitted into narrow margins of time.

Despite these pressures, experienced scholars frequently continue to provide detailed and thoughtful evaluations. Their motivation rests on the belief that their judgment contributes meaningfully to the development of their field. Yet when this labor remains largely invisible, absent from professional records and unacknowledged in institutional evaluation, it becomes difficult for reviewers to maintain a clear sense of its broader academic value.

Addressing this challenge does not require financial incentives. For many senior academics, compensation is neither expected nor particularly motivating. What is needed instead is a structured way of making evaluative contributions visible within the scholarly ecosystem.

One potential approach is the development of a reviewer recognition ledger. Such a system could record the volume, type, and general orientation of review work in a manner that allows reviewers, if they wish, to make this information publicly accessible. Alongside this, reviewers could be given the option to provide brief reflective notes (similar to judicial commentary) that situate the manuscript within ongoing disciplinary conversations. When attached to accepted publications, these annotations would offer readers additional interpretive context while also acknowledging the reviewer’s role in shaping the work’s development.

Over time, such a system would allow reviewers to establish a coherent and longitudinal record of their evaluative contributions. Profiles could illustrate areas of methodological expertise, the types of research questions a reviewer frequently engages with, and the intellectual directions they tend to support. This creates a structured way for evaluative labor to be recognized as part of a reviewer’s scholarly identity rather than remaining an unrecorded service activity.

Any system of reviewer recognition must also respect the anonymity norms that vary widely across disciplines. While some fields rely on open peer commentary, others maintain strict confidentiality to protect junior scholars and to ensure that evaluative judgment is not distorted by professional hierarchies. For this reason, recognition within the proposed model is optional and aggregated: reviewers may document their evaluative contributions without disclosing their identities or linking themselves to specific manuscripts. This approach preserves the motivational and accountability benefits of visibility while maintaining anonymity wherever it is a normative, ethical, or pragmatic requirement.

By formalizing reviewer contributions in this way, the system would support the continued engagement of experienced scholars and strengthen the transparency and coherence of evaluative processes. Recognition does not replace anonymity where it is desired, nor does it impose new hierarchies; instead, it provides an optional framework through which reviewers can make their contributions visible and meaningful within the broader architecture of scholarly communication.

## The coming tsunami: ai‑accelerated submissions

The academic publishing ecosystem is entering a period of accelerated pressure. Generative artificial intelligence has lowered the cognitive and temporal cost of producing manuscript-length text. Tools based on large language models now assist with tasks that previously constrained writing speed, including literature consolidation, argument structuring, and stylistic refinement. As these tools become embedded in research workflows, manuscript submissions are increasing across several fields, particularly in computer science, biomedicine, and applied economics, where early adoption has been most visible. This growth is uneven but persistent. It reflects a structural transition from scarcity in scientific output to rapid acceleration, a trend that places acute strain on the one element of the publishing system that has not been automated: human judgment.

Empirical evidence on AI-driven changes to desk-rejection or acceptance rates remains limited, largely because generative tools were adopted only recently and journals have not yet released systematic data on post-screening outcomes. What is already clear, however, is that large language models can now produce manuscripts with sufficient surface coherence (grammatical quality, structural organization, and rhetorical fluency) to pass editorial triage even when the underlying contribution is shallow. This shifts the burden of differentiation from editors to expert reviewers. The concern, therefore, is not documented changes in rejection statistics, but the structural inevitability that as surface quality becomes computationally cheap, the evaluative bottleneck will migrate deeper into the review pipeline.

The traditional prestige-rejection paradigm is particularly exposed in this environment. High selectivity has long served as a signal of quality, but when AI-generated manuscripts are polished, grammatically coherent, and stylistically aligned with disciplinary norms, surface quality no longer reliably indicates epistemic merit. Editors cannot rely on presentation as an index of expertise, and reviewers must distinguish between papers that only appear well-structured and those grounded in substantive insight. Prior analyses of quality-control fragility under prestige pressure suggest that even before AI, symbolic criteria complicated consistent evaluation. The introduction of automated writing tools magnifies these vulnerabilities by increasing the volume of manuscripts that meet superficial thresholds without necessarily meeting epistemic ones.

A further, emerging concern involves algorithmic submission strategies. As language models are trained on accepted papers from specific journals, they enable researchers to tailor manuscripts toward venue-specific rhetorical and structural patterns. This creates feedback loops that encourage stylistic mimicry over substantive contribution. While documented cases remain sparse, early reports of iterative AI-generated variants tested across preprint platforms suggest that optimization for acceptance may evolve into a normalized practice. In such circumstances, peer review becomes less a mechanism of critical evaluation and more an obstacle to be navigated strategically.

The central risk, however, is not a collapse of truthfulness but a collapse of integration. A rapidly expanding volume of plausible manuscripts threatens the system’s ability to connect, curate, and evaluate knowledge. The bottleneck is no longer the scarcity of research output but the scarcity of interpretive attention. Without structural reform, the archive risks shifting from a cumulative knowledge reservoir to an overcrowded repository in which meaningful contributions are difficult to identify.

In an AI-accelerated environment, the most effective way to preserve epistemic integrity is to use AI as part of the screening infrastructure itself. At the inclusion layer, journals can employ automated tools to analyze disclosure statements, detect provenance irregularities, and identify whether manuscripts follow established methodological and reporting standards. For empirical and applied studies, AI systems can reliably flag departures from conventional protocols, missing documentation, or inconsistencies that would otherwise consume reviewer time. For theoretical manuscripts, AI can assess whether the argument is built on accurate representations of existing literature and whether its internal structure follows recognized scientific principles. Crucially, these tools do not evaluate the *validity* of a new theory; they simply differentiate between manuscripts that are coherent but superficial and those that are conceptually substantive. This enables editors to route submissions according to the level of expertise required: routine or incremental work can proceed to soundness review, while high-stakes or theoretically generative claims are allocated to senior experts. By equipping editors with AI-assisted screening, the system uses computational capacity to manage computationally generated volume, ensuring that human judgment is reserved for the manuscripts that genuinely require it.

The shift from symbolic gatekeeping to epistemic resilience is essential if journals are to maintain their integrative role in an era of accelerating submissions. At the same time, implementation must remain sensitive to field-specific review norms, preserve appropriate forms of reviewer anonymity, and avoid reproducing prestige hierarchies through automated triage. The coming transformation cannot be avoided, but with appropriate architectural adjustments, it can be managed in ways that preserve the cumulative coherence of the scientific record.

## Conclusion

The academic publishing system, originally built to protect and disseminate scientific knowledge, now operates within symbolic and institutional dynamics that do not always align with epistemic goals. As examined throughout this paper, practices such as selective scarcity, prestige-oriented evaluation, and the prioritization of novelty can, in some contexts, separate contribution from recognition. Peer review, central to quality assurance, often functions under constraints that limit its capacity to support cumulative science, while reviewer labor remains largely invisible and difficult to reuse. These tendencies vary across fields, yet they reflect common structural pressures that shape how research is evaluated and categorized.

The growing influence of generative AI further intensifies these challenges by increasing submission volume and expanding the range of manuscripts that require screening. Without adjustments to evaluation workflows, the resulting strain on expert attention risks amplifying existing inefficiencies. These developments do not indicate a collapse of the system, but they do highlight the need for more scalable and transparent mechanisms to manage the accelerating flow of scholarly work.

Our analysis suggests that improvement does not require abandoning journals or peer review, but redesigning how they operate. The two proposed architectures: tiered dissemination and tiered evaluation, aim to separate methodological validation from symbolic visibility, reduce redundant review, and enable expert judgment to be applied where it is most impactful. A structured, optional framework for reviewer recognition can further support the sustainability of evaluative labor while preserving anonymity where appropriate.

The broader implications of such reforms extend beyond editorial procedures. How knowledge is evaluated and disseminated shapes professional trajectories, institutional incentives, and the distribution of scientific visibility. By rebalancing access, labor, and recognition, the proposed framework offers a pathway toward a more resilient and transparent academic ecosystem; one that accommodates field-level diversity while strengthening the conditions for cumulative science.

Scientific progress depends not only on the discovery of new results, but on the reliability, inclusiveness, and interpretability of the archive that records them. As content production accelerates and evaluative demands grow more complex, aligning publication infrastructure with epistemic values becomes increasingly necessary. The models presented here are intended as feasible conceptual tools for supporting that alignment and for guiding the evolution of scholarly communication in a rapidly changing research landscape.

## Data Availability

No data was used for this production.
